# VariED: the first integrated database of gene annotation and expression profiles for variants related to human diseases

**DOI:** 10.1093/database/baz075

**Published:** 2019-07-17

**Authors:** Chien-Yueh Lee, Amrita Chattopadhyay, Li-Mei Chiang, Jyh-Ming Jimmy Juang, Liang-Chuan Lai, Mong-Hsun Tsai, Tzu-Pin Lu, Eric Y Chuang

**Affiliations:** 1 Graduate Institute of Biomedical Electronics and Bioinformatics, National Taiwan University, Taipei, Taiwan; 2 Bioinformatics and Biostatistics Core, Center of Genomic Medicine, National Taiwan University, Taipei, Taiwan; 3 Cardiovascular Center and Division of Cardiology, Department of Internal Medicine, National Taiwan University Hospital, Taipei, Taiwan; 4 College of Medicine, National Taiwan University, Taipei, Taiwan; 5 Graduate Institute of Physiology, National Taiwan University, Taipei, Taiwan; 6 Institute of Biotechnology, National Taiwan University, Taipei, Taiwan; 7 Center for Biotechnology, National Taiwan University, Taipei, Taiwan; 8 Institute of Epidemiology and Preventive Medicine, National Taiwan University, Taipei, Taiwan; 9 Department of Surgery, National Taiwan University Hospital, Taipei, Taiwan

## Abstract

Integrated analysis of DNA variants and gene expression profiles may facilitate precise identification of gene regulatory networks involved in disease mechanisms. Despite the widespread availability of public resources, we lack databases that are capable of simultaneously providing gene expression profiles, variant annotations, functional prediction scores and pathogenic analyses. VariED is the first web-based querying system that integrates an annotation database and expression profiles for genetic variants. The database offers a user-friendly platform and locates gene/variant names in the literature by connecting to established online querying tools, biological annotation tools and records from free-text literature. VariED acts as a central hub for organized genome information consisting of gene annotation, variant allele frequency, functional prediction, clinical interpretation and gene expression profiles in three species: human, mouse and zebrafish. VariED also provides a novel scoring scheme to predict the functional impact of a DNA variant. With one single entry, all results regarding queried DNA variants can be downloaded. VariED can potentially serve as an efficient way to obtain comprehensive variant knowledge for clinicians and scientists around the world working on important drug discoveries and precision treatments.

## Introduction

Advances in microarray and next-generation sequencing (NGS) technologies and the widespread availability of genomic data provide a good opportunity for researchers to simultaneously analyze data from multiple molecular levels. Knowledge of detailed variant information along with corresponding gene expression profiles for specific organs is considered a key step for diagnostic and prognostic assessment of the functional effects of genetic variants. Therefore, establishing a comprehensive analytical system that provides multi-level information for a DNA variant may greatly benefit researchers.

The surge in analysis of gene expression data in recent years has led to the development of various web-based tools and databases that help to explore the genetic roots of diseases in different populations (1, 2). For example, databases such as ANNOVAR (3), InterVar (4), FUMA (5), Ensembl (6) and UCSC (7) provide gene annotation information, and databases such as ProteinAtlas (8), GeneCards (9), NCBI (10) and UniProt (11) furnish tissue-specific gene expression profile information. However, no tools or databases exist that are capable of simultaneously providing (i) gene expression profiles, (ii) variant annotations, (iii) functional prediction scores and (iv) pathogenic analyses simultaneously. Moreover, certain limitations exist in each of the existing databases. Some databases fail to conduct multigene query, whereas others allow a limited number of DNA variants for batch gene query (8, 9). Other online databases, such as 1000 Genomes (12), Exome Aggregation Consortium (ExAC) (13), NHLBI Exome Sequencing Project (ESP) (14), Integrative Japanese Genome Variation Database (IJGVD) (15), Taiwan Biobank (TWB) (16) and genome Aggregation Database (gnomAD) (17), provide allele frequencies of DNA variants but only allow searches of one gene or variant at a time (8). Therefore, we still lack a comprehensive analytical system that integrates different kinds of information, such as the functional impact of DNA variants, allele frequencies in different populations and gene expression levels in specific tissue types.

In this study, we present VariED, a user-friendly web-based system with an integrated annotation database for human genetic variants. It is the first integrated database system that balances the pros and cons of the existing databases by linking comprehensive multi-level knowledge of a DNA variant and its corresponding gene expression profiles from established online tools through three molecular levels—DNA, RNA and protein—in human, mouse and zebrafish. It further offers an unlimited search option with easily interpretable detailed tabular outputs. While conducting functional assays, expression labels from some organs (e.g., heart) might not be available for humans; hence, model organisms such as mouse might serve as an alternate source for human expression labels. For medical practitioners it is especially challenging to locate such alternate expression labels. To the advantage of such users and others, VariED provides integrated cross-species variant annotation and gene expression labels, derived from the two most important model organisms, zebrafish and mouse, using orthologous genes as the link between different species. Such organized information would not only improve the efficiency of analyses but also facilitate dissecting the biological mechanism of specific diseases. With a single entry, all important information regarding queried DNA variants is downloadable in VariED.

## Material and Methods

### Overview of VariED workflow

**Figure 1:**
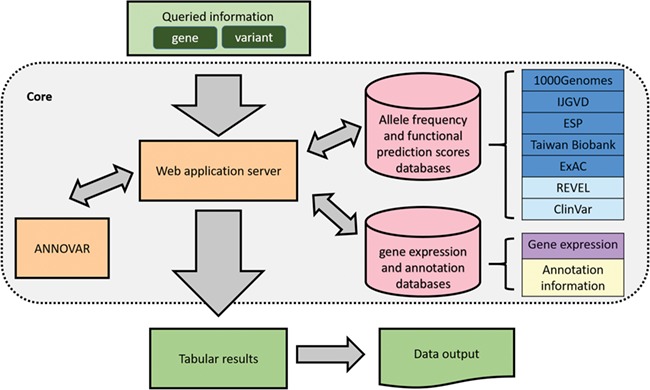
Overview of VariED database.

An overview of VariED is illustrated in Figure 1. It is a broadly accessible data collection and processing platform, consisting of variant information that is mined and integrated from existing online resources. Table 1 displays a comprehensive summary of VariED with a point-by-point comparison of its available features with respect to different existing databases. Only VariED offers an integrated platform comprising both gene expression information and functional effects of variants (Table 1), thus acting as a source for efficient and systematic genome information. It contains variant information such as gene annotation, population allele frequency, gene expression profiles, functional prediction and clinical interpretation from human (*Homo sapiens*), mouse (*Mus musculus*) and zebrafish (*Danio rerio*). VariED further proposes a unique ‘index score’, to predict the pathogenicity of the queried variants. In this study, we introduce the VariED system, providing a thorough description of its features. The database was developed by using Flask framework with Python 3.4 and MySQL. VariED offers an unlimited search option where users can key in gene or variant names (single or multiple) to obtain easily interpretable tabular outputs that are downloadable in csv format.

**Table 1:** Comparison of functions and query results offered by existing databases

	Population allele frequency						GE	VCF file	Batch search	Functional prediction scores			Clinical interpretation
	1000 Genomes	ESP	IJGVD	TWB	ExAC	gnomAD				REVEL	GERP++	CADD	prediction
VariED	+	+	+	+	+	+	+	+	+	+	+	+	+
ANNOVAR	+	+			+	+		+	+	+	+	+	
InterVar	+	+			+			+/−^a^	+/−^a^		+		+
ProteinAtlas							+						
HGMD								+/−^b^	+/−^b^		+/−^b^		
Uniprot							+/−^c^		+				
GeneCard							+		+/−^d^				
FUMA	+						+					+	
UCSC	+				+	+	+	+	+		+		
HaploReg	+								+		+		
NCBI	+				+	+	+						+
Ensembl	+	+			+	+		+	+	+		+	+

Notes: ESP, NHLBI Exome Sequencing Project; IJGVD, Integrative Japanese Genome Variation Database; TWB, Taiwan Biobank; ExAC, Exome Aggregation Consortium; GE, gene expression; REVEL, Rare Exome Variant Ensemble Learner; GERP, Genomic Evolutionary Rate Profiling; CADD, Combined Annotation Dependent Depletion; +, complete information support; +/−, partial information support.

^a^Script version only.

^b^Professional version only.

^c^Simple declaration only.

^d^100 genes per query or pay for getting an annual unlimited license.

### Database contents and construction

Presently VariED has information content for more than 709 million variants, including common, rare, intronic and non-coding variants that have been integrated from multiple existing sources such as NHLBI ESP (14), 1000 Genomes Project (12), ExAC (13), IJGVD (15), TWB (16) and gnomAD (17). VariED primarily contains information for DNA variant and gene expression profiles from various organs of human (reference genome: GRCh37p.13/GRCh38p.12), mouse (reference genome: GRCm38.p6) and zebrafish (reference genome: GRCz11). Human genome versions GRCh37 and GRCh38 are supported in the ‘Expression Profiles’ function of VariED; however, the system currently works with annotation data of variants on only GRCh37. VariED will fully support GRCh38 in the next updated release. Sources of population allele frequency information are NHLBI ESP (14), 1000 Genomes Project (12), ExAC (13), IJGVD (15), TWB (16) and gnomAD (17). NHLBI ESP consists of well-phenotyped populations (African American and European American) from the United States with more than 200 000 individuals altogether. The 1000 Genomes phase 3 data includes populations such as East Asians, Americans, Africans, Europeans and South Asians. ExAC consists of jointly analyzed exome data from nearly 92 000 individuals, with a publicly accessible data set spanning 61 486 of these individuals for use as a global reference set. IJGVD is a Japanese population reference panel providing variant allele frequencies from 1KJPN and 2KJPN. TWB provides allele frequencies for the Taiwanese population using microarray and NGS data from 21 695 and 1517 individuals, respectively. GnomAD consists of allele frequency information from 123 136 exomes and 15 496 genomes from Latino, African, Ashkenazi Jewish, European, South Asian and East Asian ethnic populations. Gene expression profiles are collected or estimated from The Human Protein Atlas (8), Expression Atlas (18) and NCBI SRA (19). Supplementary Information describes the details of gene expression data in VariED. Moreover, VariED proposes a novel ‘index score’ and further accesses CADD scores (20), REVEL scores (21) and GERP++ (22) scores to predict the pathogenicity of the variants. It also utilizes ANNOVAR (3) and ClinVar (23) to include functional annotation and clinical significance, respectively, for user-queried genetic variants. A detailed description of release versions of VariED incorporated databases and tools are provided in Table S1.

To understand gene expression profiles of humans against model species, orthologous genes in human, mouse and zebrafish are collected from the HUGO Gene Nomenclature Committee (HGNC). Comparison of Orthology Predictions (24) that integrates orthology predictions from 14 databases. Orthologous genes supported by less than three databases were excluded, resulting in a total of 18 690, 19 037 and 18 268 genes in human, mouse and zebrafish, respectively.

One of the challenges associated with querying variants is the inconsistency of gene names across different species. For example, *COX*, *COX8*, *COX8-2* and *COX8L* could be the aliases of the same gene, *COX*, from different species. To circumvent this ambiguity, VariED unifies gene symbols from Ensembl gene symbol (6), NCBI gene symbol (10) and NCBI aliases (10) and searches queried variants in the specified order. For such scenarios, VariED uses orthologous gene IDs to search for the gene in different species and their corresponding tissues, as specified by the user.

### Prediction of variants’ functions

For predicting the function of genetic variants, VariED accepts variant call format (VCF) files or a list of variants (e.g. chr1:69224A > T) as input from users. While querying the database, the users can choose from a variety of options pertaining to variants, population of interest and tissue or organ under focus. This version provides links to 1000 Genomes, IJGVD, NHLBI ESP, TWB, ExAC and gnomAD as source options for the reference population. Gene expression profile score, TPM, is used to report the expression of a variant of a tissue of interest. A threshold of TPM (> 0.5) is used to report expression and is displayed through ‘Yes’ otherwise ‘No’. Functional prediction does not completely explain the clinical interpretation of the variants. Keeping that in view, the functional interpretation tab provides a unique ‘index score’ that has been introduced in VariED to predict the functional consequence of the variant(s). The index score is based on the type of variant, the REVEL score, the GERP++ score and the gene expression profiles in the target tissue in humans. The index score classifies a variant based on its mutational properties, its position in the genome and its functional role in specific diseases. An index score of 0 implies that the variant is not pathogenic (Table 2), a prediction based on genomic annotation (e.g. intronic, intergenic or synonymous variant). An index score of 1 signifies the variant to be non-synonymous or low precedence or that its corresponding REVEL score is less than a specified threshold (default threshold is 0.5), suggesting less pathogenicity. If a variant is of high precedence or in the splice site or if the variant is of low precedence and has a score greater than the fixed threshold, the variant will display an index score of 2, implying moderate pathogenicity. For higher specificity the users can set the threshold to 0.75. If a variant meets the requirements for an index score of 2 and the gene expression of the variant meets the criteria of TPM > 0.5 or the GERP++ score of this variant is more than 2, it will be assigned an index score of 3, inferring the variant to be highly pathogenic. Other functional prediction scores such as CADD (20), REVEL (21) and GERP++ (22) imported from existing data sources are also integrated in VariED. The reason for reporting multiple scores is to provide validation of each individual score and aid users with a comparative analysis of the protein products of each variant. Furthermore, ClinVar (23) is integrated into our system to provide the clinical significance of the variant(s). Such information retrieval is a valuable and facile method for obtaining knowledge about human diseases. The integrated results will be split into four parts: (i) gene annotation, (ii) population allele frequency, (iii) functional prediction and (iv) clinical interpretation.

**Table 2:** Indices and their functional consequences


**Index**	**Condition**	**Functional consequence**
0	Intronic or intergenic or Synonymous	Non-pathogenic
1	Low precedence^a^ or REVEL score < threshold^b^	Less-pathogenic
2	High precedence^c^ or splicing or (low precedence & REVEL score > threshold^b^)	Moderately pathogenic
3	Index = 2, and (expression value (TPM) > = 0.5 or GERP++ score > 2)	Highly pathogenic

^a^Low precedence: non-frameshift insertion, non-frameshift deletion, non-frameshift substitution and nonsynonymous SNV.

^b^Threshold = 0.5 (default), for higher specificity the threshold can be set to 0.75.

^c^High precedence: frameshift insertion, frameshift deletion, frameshift substitution, stop-gain, stop-loss.

### Prediction of gene expression profiles

VariED accepts gene symbols or Ensembl gene IDs to provide gene annotation and tissue-specific gene expression profiles of the desired input. The search enables the users to view differently categorized and annotated genes and expression profiles through pre-defined options that link the search to a user-specified species (human, mouse or zebrafish), along with an option to switch between genome builds (GRCh37 and GRCh38) in humans. Just like the variant information, all expression profiles can be downloaded in csv format.

## Results

### Website interface

VariED offers two main functions: the ‘Variants search’ tab provides the gene description, allele frequency, functional prediction scores and clinical interpretation, and the ‘Expression profiles’ tab offers users the option to query gene annotations and tissue-specific gene expression profiles. The size of the database is 33.81 GB, and it houses information for approximately 709 million variants. The average time to query 10 k variants for all information would be approximately 17 min.

### Example 1: using the ‘variants search’ function for gene annotation information and finding pathogenic variants

**Figure 2:**
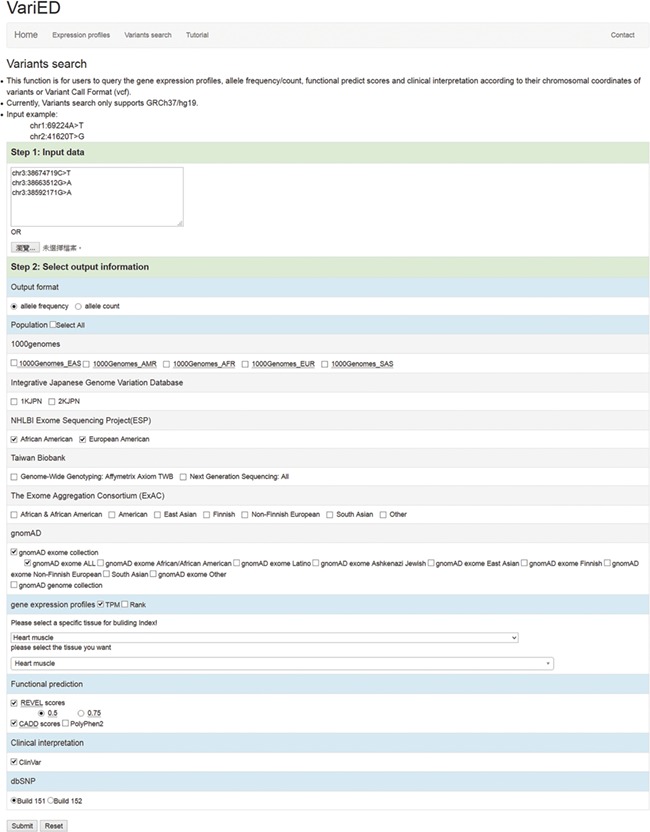
Screenshot of the ‘Variants search’ input page. This page is used to (i) input user queried variants (using chromosomal coordinates or VCF files) and (ii) select output information including allele frequency/counts, reference populations (1000 Genomes/IJGVD/ESP/TWB/ExAC/gnomAD), tissue of interest (any), gene expression profile score (TPM or Rank), functional prediction scores (REVEL, CADD and PolyPhen2), clinical interpretation (ClinVar) and dbSNP versions (Build 151 or Build 152). These options dictate the gene description, allele frequency, functional prediction and clinical interpretation for one or more user-queried variants.

**Figure 3:**
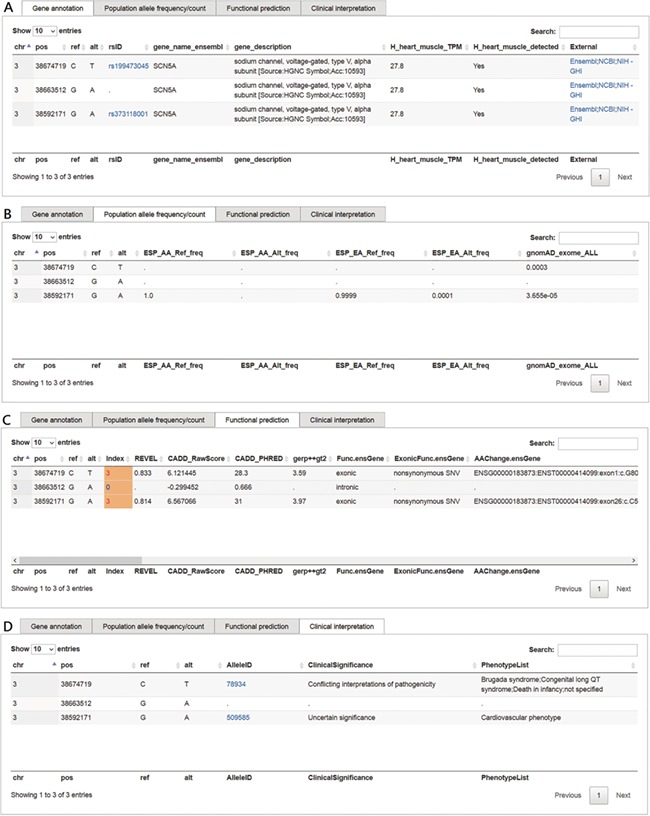
Screenshots of outputs from the ‘Variants search’ function. (**A**) Gene annotation and description of the queried variant, the ‘rsID’ column provides a hyperlink to dbSNP; the ‘External’ column provides hyperlinks to Ensembl, NCBI gene and NIH Genetics Home Reference (NIH—GHI). (**B**) Allele frequencies of queried variants for each of the chosen reference populations. (**C**) Functional prediction along with scores such as VariED index, REVEL, CADD_raw, CADD_Phred and GERP++. (**D**) Clinical significance of the queried variant, the ‘AlleleID’ column provides a hyperlink to ClinVar.

This function in VariED is for users to input variant specifics to obtain annotation information and identify pathogenic variants. Figure 2 shows a screenshot of the ‘Variants search’ page with an example of three variants from *SCN5A* (25) with their chromosomal coordinates used as input. Users can query such multiple variants from a single gene or multiple genes using chromosomal coordinates (e.g., chr3:38674719C>T, chr3:38663512G>A and chr3:38592171G>A) (Figure 2) or by uploading VCF files. They can further select the reference population and the tissue of interest as parameters for acquiring the specified gene annotations (Figure 2). VariED outputs a description of the genes (Figure 3A), allele frequency or allele count (Figure 3B), functional prediction (Figure 3C) and clinical interpretation (Figure 3D) of the queried variants. The output displays the index score of three DNA variants (3:38674719C>T, 3:38663512G>A and 3:38592171G>A) (Figure 3C). Variant 3:38674719C>T is assigned an index 3, indicating strong evidence for its pathogenicity and re-affirming it as an important biomarker for Brugada syndrome, whereas variant 3:38663512G>A is assigned an index 0, confirming its non-pathogenicity. The index score is inferred based on the type, REVEL score and GERP++ score of each variant. Further, it provides an additional view of the variant under query through its gene expression levels in the target tissue. Coupling of these two features is advantageous when compared to using each feature alone, as it leads to prioritization of candidate targets, thus significantly enhancing the predictive power of the variant under query. To sum up, the index score incorporates all aspects of the functional consequences of a variant along with its pathogenicity by taking into account the expression profile of the variant under study with respect to specific diseases. CADD is not used to produce the index score, so VariED simply imports the CADD score values (Figure 3C), thus providing a comprehensive functional interpretation of the query to the user. A correlation analysis using all variants from NHLBI ESP data set reveals that the index score in VariED has moderate correlations (Kendal’s Tau) of 0.46 (r) with both the CADD_RawScore and CADD_Phred (Table S2). A comparative analysis of other scores (REVEL and GERP++) with CADD shows that the correlations of REVEL and GERP++ with CADD are 0.47 (r) and 0.35 (r), respectively (Table S2). Even though the correlation of REVEL with CADD is marginally higher than that of the index with CADD, the difference is not significant. Furthermore, the entire ClinVar data set is applied to determine the predictive ability of index score. The variants from the ClinVar data set are divided into benign (*n* = 5034) and pathogenic (*n* = 1119) groups. The sensitivity, specificity and accuracy are analyzed using a suggested cutoff value for each data set. The results suggest that the index score performs at par or better than other scores, especially the sensitivity and accuracy of index score are significantly higher than GERP++ and CADD scores alone. Table S3 displays comparison results for the index, CADD, GERP++ and REVEL scores based on the ClinVar variants. Moreover, the gene expression information in the index score provides a better understanding of genes for experimental validations. The CADD_RawScore scores deleteriousness of variants, indicating them to be simulated (or ‘not observed’) and therefore more likely to have deleterious effects. CADD_Phred defines the rank for each variant within a specific group of variants. REVEL (21) scores predict the pathogenicity of missense variants, and GERP++ (22) scores from ANNOVAR (3) predict the evolutionary conservation of variants. The clinical significance (Figure 3D) of the variants is obtained through ‘Allele ID’, where ‘Clinical Significance’ and ‘Phenotype List’ provide users with information on the relationships among human variations and phenotypes.

### Example 2: using the ‘gene expression profiles’ function to filter the candidate gene in heart diseases

**Figure 4:**
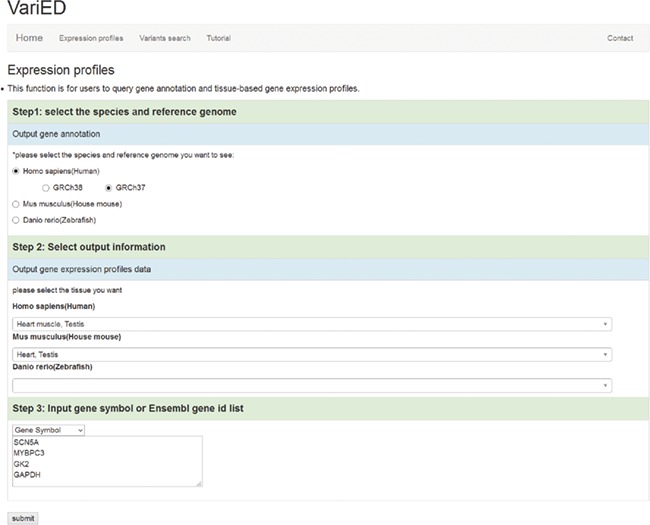
Screenshot of the ‘Expression profiles’ page. This page is used to (i) input user-specified gene names, (ii) select reference populations/species and (iii) indicate the tissue/organ of interest for obtaining gene expression profiles.

**Figure 5:**
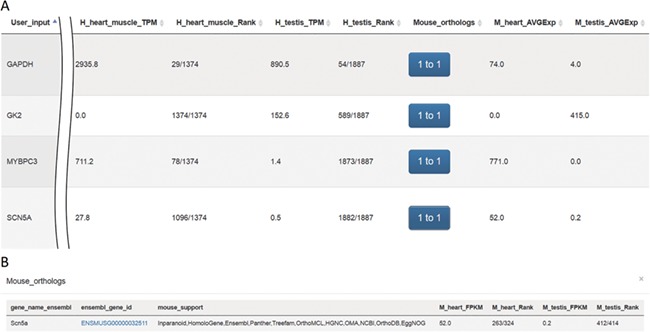
Screenshots of outputs from the ‘Expression profiles’ function. (**A**) Gene expression profiles for heart and testis in human and mouse for genes *SCN5A*, *MYBPC3*, *GK2* and *GAPDH.* (**B**) Mouse orthologs for the *SCN5A* gene.

VariED integrates gene expression profile information from all tissues of three species (human, mouse and zebrafish). With the integrated gene expression information, users can filter candidate genes to confirm those with expression in a specified tissue (e.g. heart tissue). VariED uses orthologous gene IDs to search for the queried gene in different species and their corresponding tissues, as specified by the user (Figure 4). The queried gene symbol is searched in Ensembl (6) or NCBI (10), and if found in neither, VariED searches for its aliases to provide the user with options to select the exact gene name as the desired input, through an intermediate page. Finally, based on the user-specified reference genome, species and tissue(s), a cross-species search would display one or more gene annotations (Figure 5A) in tabular format.

The system was tested using gene symbols *SCN5A* (25), *GAPDH* (26), *GK2* (27) and *MYBPC3* (28) (Figure 5A). The protein encoded by gene *SCN5A* is found primarily in cardiac muscle and is responsible for the initial upstroke of the action potential in an electrocardiogram. *GAPDH* encodes a member of the glyceraldehyde-3-phosphate dehydrogenase protein family. It is a glycolytic enzyme that is affected during heart failure and is expressed in testis. Also, *GAPDH* is one of the housekeeping genes most commonly used in comparisons of gene expression data. The protein product of *GK2* (glycerol kinase 2) is localized in the outer membrane of the mitochondrion and is expressed at high levels in testis but not in heart tissue. *MYBPC3*, a cardiac isoform, is expressed exclusively in heart muscle. Results queried using VariED support hypothesized functions for all four genes (Figure 5A), thereby assisting users in selecting candidate genes with expression in specific tissues (e.g. heart) and furnishing orthologs in model organisms such as mouse (Figure 5B) or zebrafish.

## Discussion and Conclusions

To exploit the ongoing development of high-throughput sequencing innovations and enormous public resources, VariED provides a complete expository framework of gene annotation and gene expression profiles for approximately 709 million DNA variants. Most of the numerous other tools and methodologies that have been developed to analyze such multi-level data are unable to provide all facets of the queried DNA variants through a single system alone (Table 1), which gives VariED an edge over similar existing tools. Moreover, VariED predicts the functional impact of queried DNA variants using a novel index score that is composed of several popular prediction algorithms and scores (REVEL and GERP++), the expression profile of the variant in the target tissue and associated clinical characteristics, thus furnishing users with a ready-made multi-dimensional view of the query.

Example 1 displays the index score of three DNA variants (3:38674719C>T, 3:38663512G>A and 3:38592171G>A) (Figure 3C). The findings suggest that the index score calculated in VariED can serve as an efficient selection tool to rank the possibility of queried variants playing an important role in a biological process (Figure 3D). Furthermore, VariED provides a strategy by which users can prioritize important DNA variants based on a gene query. On conducting a search in VariED using a gene entry, a list of variants from queried genes is displayed, along with their functional prediction scores and clinical significance. As it is challenging to conduct functional assays on such a large number of variants, VariED comes in handy, as it displays the variants in order of their importance based on their respective prediction scores and expression rates in tissues or organs of interest. For example, VariED assigned an index score of 3 to the variant 3:38592171G>A (entry 3 in Figure 2), an exonic variant from *SCN5A* reported to be associated with cardiovascular diseases (29), thus implying the variant’s potential pathogenicity. Based on such strong evidence (index = 3), users can prioritize variant 3:38592171G>A as an important player for the disease. We also provide an evaluation of the index score by comparing the classification of DNA variants in our index scoring system with the CADD system based on the variants from the ESP. A moderate positive correlation is observed between the index score and both CADD raw and CADD phred-like scores (*r* = 0.46 and 0.46, respectively) using the entire set of (1 982 300) variants (Table S2). Furthermore, a comparative analysis of the index score (Table S3) using the entire ClinVar data set displayed it to perform better than CADD, REVEL and GERP++ scores.

The expression profiles from mouse and zebrafish are included in VariED because they are popular model species for human disease research. However, VariED currently only contains gene expression profiles from the species in the adult stage, such as adult male C57BL/6 mouse expression profiles, which might pose limitations in analyzing the functional impact of DNA variants in an embryo. Moreover, the gene expression profiles of mouse and zebrafish were obtained from only one RNA-sequencing experiment, which might result in identification of false positive DNA variants due to systematic bias. To address such shortcomings, the human gene expression profiles in VariED were derived from nine replicates of RNA-sequencing data. In the future, we aim to include expression profiles from different developmental stages using replicates of sequencing data for animal models as well.

The continuous increase of data in genomics demands import strategies that needs to be employed in the future. With the current architecture of VariED, the implemented database does not have enough scalability to deal with it. Until now, several query and storage engines, such as Apache Drill (https://drill.apache.org), Hive (https://hive.apache.org), Impala (http://impala.io), Kylin (http://kylin.apache.org), Spark (https://spark.apache.org), Presto (https://prestodb.github.io), etc. have been proposed for big data applications. Some of them have been successfully applied in recent genomic studies and have shown significant improvement in the performance (30, 31). Going forward, VariED will incorporate such engines to cope with the continued dramatic growth in the volume of genomic data to be acquired for the next few years.

The primary goal of our work is to elevate the user’s experience while using our system to extract organized information on genes and variants from various web and literature sources. VariED is a tool that would help clinicians by making available, for the first time, a comprehensive panel of information encompassing both clinical characteristics and biological factors that would lead to easier identification of the right treatment for the right patient. Traditionally, diagnosis and treatment recommendations are made without any reference to variability of linked genes. Precision medicine demands targeted drug treatment and prevention of diseases, taking into account the complex interplay of clinical phenotypes, genes and variants. In the current wave of genetic and high-throughput information that is available and ready to be utilized, VariED could serve as a central hub of comprehensive variant knowledge, to help clinicians and scientists around the world with important drug discoveries and precision treatments.

## Supplementary Material

Supplementary_Information_baz075

Table_S1_updated_baz075

Table_S2_baz075

Table_S3_baz075

## Notes

**Database URL**: http://varied.cgm.ntu.edu.tw/
