# Supplementary material for: VariED: the first integrated database of gene annotation and expression profiles for variants related to human diseases
Source: Database (Oxford). 2019 Jul 17;2019:baz075. doi: 10.1093/database/baz075 (PMC6637258; doi:10.1093/database/baz075)
Supplement: Supplementary_Information_baz075 [file supplementary_information_baz075.doc]

Supplementary Information

**Gene Expression data in VariED**

The gene expression data of human was collected as TPM (Transcripts Per Kilobase Million) directly as “rna_tissue.tsv” downloaded from the Human Protein Atlas. For Mouse, gene expression data, only as FPKM (Fragments Per Kilobase of exon per million fragments mapped) were downloaded from Expression Atlas (E-GEOD-74747). The gene expression data of human and mouse were downloaded without any further processing. The RNA-seq dataset of zebrafish with accession numbers SRX1153632, SRX1153633, and SRX1153634 were collected from NCBI SRA. For alignment, all the three RNA-seq samples were aligned to GRCz10 version of zebrafish genome using Tophat2 (v2.1.1). Then, Cufflinks (v2.2.1) was utilized to calculate FPKM values. Currently, VariED uses both FPKM and TPM. However, TPM is used only for gene expression data of humans, and FPKM is used for mouse and zebrafish. The VariED output from “Variants search” includes both TPM and "detected" columns for the chosen tissues. Gene expression profile score of TPM is used to report the expression of a variant of a tissue of interest. A threshold of TPM is used to report expression or not (Yes/No) in the tissue under query. In this report a TPM>=0.5 is used to report gene expression as detected (“Yes”) and TPM<0.5 reports gene expression as not detected (“No”) in this study.
