# Supplementary material for: VariED: the first integrated database of gene annotation and expression profiles for variants related to human diseases
Source: Database (Oxford). 2019 Jul 17;2019:baz075. doi: 10.1093/database/baz075 (PMC6637258; doi:10.1093/database/baz075)
Supplement: Table_S1_updated_baz075 [file table_s1_updated_baz075.docx]

**Table S1.** Release versions of VariED incorporated tools and databases

| Source | Release Version | Download Date |
| --- | --- | --- |
| Gene Annotation | Ensembl Release 96 | 2019/05/04 |
| Gene Aliases (NCBI gene info) |  | 2019/05/04 |
| dbSNP | Release Version 151 | 2017/10/06 |
| dbSNP | Release Version 152 | 2018/10/25 |
| The Human Protein Atlas | Protein Atlas ver. 18.1 | 2019/05/04 |
| Expression Atlas (E-GEOD-74747) |  | 2019/05/04 |
| 1000 Genomes Project Phase 3 |  | 2016/09/27 |
| NHLBI GO Exome Sequencing Project |  | 2016/05/25 |
| Tohoku Medical Megabank Project (TMM) | 1KJPN, 2KJPN |  |
| Taiwan Biobank (Microarray) |  | 2017/03/03 |
| Taiwan Biobank (NGS) |  | 2017/12/21 |
| REVEL |  | 2016/06/03 |
| CADD | Release v1.3 |  |
