# Supplementary material for: VariED: the first integrated database of gene annotation and expression profiles for variants related to human diseases
Source: Database (Oxford). 2019 Jul 17;2019:baz075. doi: 10.1093/database/baz075 (PMC6637258; doi:10.1093/database/baz075)
Supplement: Table_S2_baz075 [file table_s2_baz075.doc]

| **Table S2.** The CADD score and its comparative correlation analysis with the index, REVEL, and GERP++ scores based on NHLBI ESP variants | | | |
| --- | --- | --- | --- |
|  | Index | REVEL | GERP++ |
| **CADD_Rawscore** | r = 0.46  n = 1,982,300 | r = 0.47  n = 735,203 | r = 0.35  n = 774,348 |
| **CADD_PHRED** | r = 0.46  n = 1,982,300 | r = 0.47  n = 735,203 | r = 0.35  n = 774,348 |
