# Supplementary material for: VariED: the first integrated database of gene annotation and expression profiles for variants related to human diseases
Source: Database (Oxford). 2019 Jul 17;2019:baz075. doi: 10.1093/database/baz075 (PMC6637258; doi:10.1093/database/baz075)
Supplement: Table_S3_baz075 [file table_s3_baz075.doc]

| **Table S3.** Comparison results of the predictive ability for the index, CADD, GERP++, and REVEL scores based on the ClinVar variants | | | | | | |
| --- | --- | --- | --- | --- | --- | --- |
| **Dataset and its suggested cutoff value** | **Benign** | **Pathogenic** | **Total** | **Sensitivity** | **Specificity** | **Accuracy** |
| Index <= 1 | 4,694 | 269 | 4,963 | 0.714 | 0.946 | 0.901 |
| Index > 1 | 340 | 850 | 1,190 |
| REVEL < 0.5 | 4,692 | 284 | 4,976 | 0.709 | 0.943 | 0.898 |
| REVEL >= 0.5 | 342 | 835 | 1,177 |
| GERP++ < 2 | 2,099 | 117 | 2,216 | 0.255 | 0.947 | 0.504 |
| GERP++ >= 2 | 2,935 | 1,002 | 3,937 |
| CADD_PHRED < 15 | 2,832 | 110 | 2,942 | 0.314 | 0.963 | 0.624 |
| CADD_PHRED >= 15 | 2,202 | 1,009 | 3,211 |
